# The positive short‐term effect of dexamethasone on ataxia symptoms in a patient with ataxia‐telangiectasia: A case report

**DOI:** 10.1002/ccr3.5895

**Published:** 2022-05-20

**Authors:** Maryam Saberi‐Karimian, Mehran Beyraghi‐Tousi, Tannaz Jamialahmadi, Amirhossein Sahebkar

**Affiliations:** ^1^ 37552 Vascular and Endovascular Surgery Research Center Mashhad University of Medical Sciences Mashhad Iran; ^2^ 37552 Surgical Oncology Research Center Mashhad University of Medical Sciences Mashhad Iran; ^3^ 37552 International UNESCO center for Health Related Basic Sciences and Human Nutrition Mashhad University of Medical Sciences Mashhad Iran; ^4^ 37552 Department of Pediatric Diseases Faculty of Medicine Mashhad University of Medical Sciences Mashhad Iran; ^5^ 37552 Biotechnology Research Center Pharmaceutical Technology Institute Mashhad University of Medical Sciences Mashhad Iran; ^6^ 37552 Applied Biomedical Research Center Mashhad University of Medical Sciences Mashhad Iran; ^7^ School of Medicine The University of Western Australia Perth Australia; ^8^ 37552 Department of Biotechnology School of Pharmacy Mashhad University of Medical Sciences Mashhad Iran

**Keywords:** allergy, ataxia‐telangiectasia, cerebral palsy, dexamethasone, physical therapy

## Abstract

Oral dexamethasone was administered at a dose of 0.075 mg/kg/day for a boy with ataxia‐telangiectasia. The SARA score was improved by 7.0 points after dexamethasone treatment over a period of 28 days. The body weight was increased by 1.4 kg after 4 weeks leading to dose titration to 0.05 mg/kg/day.

## Graphical abstract

AT is a rare neurodegenerative genetic disorder caused by biallelic mutations in the AT‐mutated gene. Glucocorticoid analogs can improve neurological symptoms in AT patients. Taking oral dexamethasone at a dose of 0.075 mg/kg/day for 28 days can improve ataxia symptoms in AT patients.

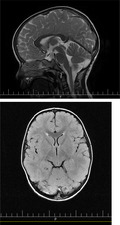

## 1 BACKGROUND

Ataxia‐telangiectasia (AT) is a rare neurodegenerative genetic disorder caused by biallelic mutations in the AT‐mutated (ATM) gene. ATM encodes for a protein kinase belonging to the phosphoinositide 3‐kinase (PI3) family.^1^

Patients with AT display a complicated phenotype, including an early‐onset progressive cerebellar ataxia, oculocutaneous telangiectasias, immunodeficiency, high incidence of pulmonary infections, a tendency to cancers and radio sensitivity.^2^, ^3^, ^4^ Their lifespan is around 25 years.^5^

Unfortunately, there is no therapy currently available for this disorder. However, previous studies showed that short‐term treatment with glucocorticoid (GCs) analogs can improve neurological symptoms in AT patients.^6^, ^7^ Leuzzi et al. developed a method to overcome the corticosteroid‐induced side effects by a long‐term erythrocyte‐delivered dexamethasone (Ery‐Dex) treatment.^8^ Ery‐Dex was reported to significantly improve neurological symptoms without the typical steroids side effects.^8^

Due to the lack of immunodeficiency and since Ery‐Dex is not available in the market yet, the patient in the present study was administered dexamethasone.

## 2 CASE PRESENTATION

The present case report involves a boy of Iran nationality, suffering from AT, aged 6 years (height 118 cm and weight 20.5 kg) without any AT family history at least in his last 3 generations. However, his parents had a consanguineous marriage—first cousin. The parents signed the informed consent form.

## 3 SYMPTOMS AND DIAGNOSIS

This child had very severe colic in infancy. By the time he started walking at 11th month, the baby was walking on tiptoe and on its toes. The pediatric neurologist diagnosed mild cerebral palsy (CP).

**FIGURE 1:**
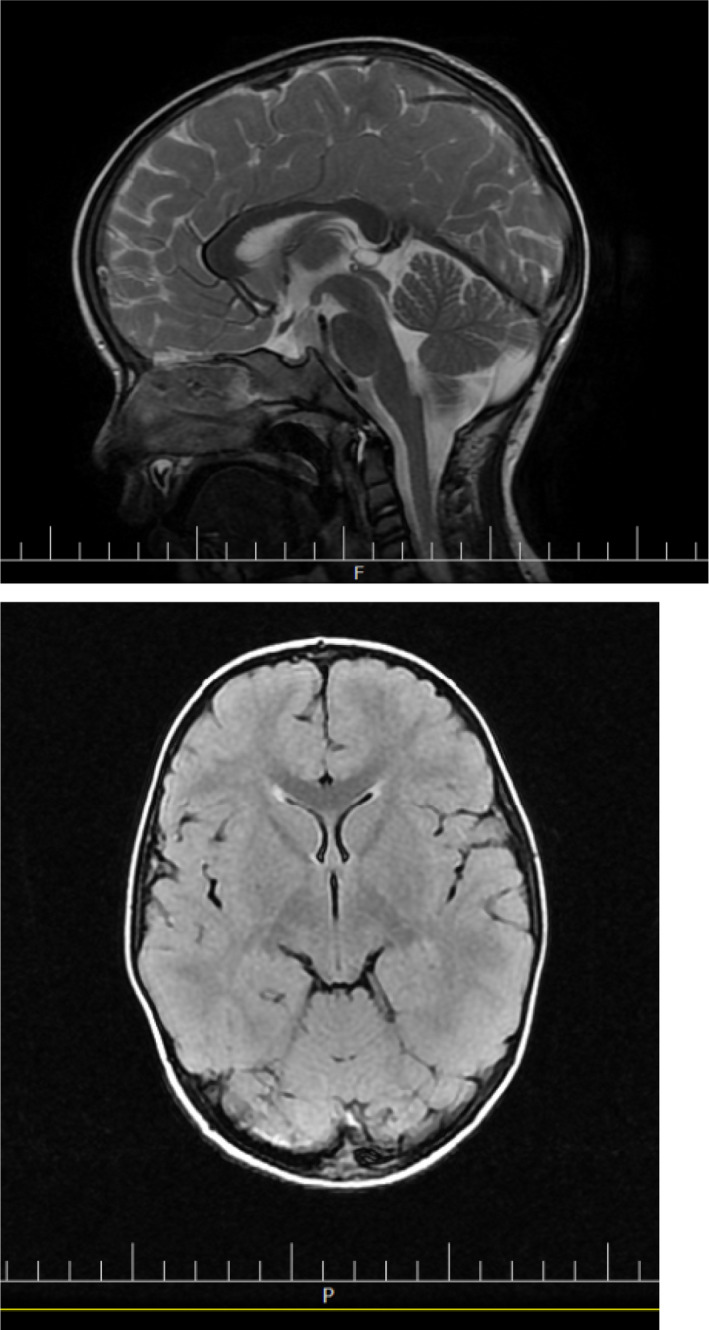
Brain MRI in a subject with ataxia‐telangiectasia when aged 2 years old

At the age of 2 years, he developed oral herpetic lesions and he could not eat for several days due to severe painful plagues, thus leading to weight loss. After recovering, his gait worsened, with him falling down every 4 steps he walked, leaving the sole of his left foot inward. Upon parental referral to a pediatric neurologist, brain MRI (Figure 1), and tandem mass spectrometry (MS/MS) tests were prescribed, all of which showed normal results and the physician again diagnosed mild CP.

**FIGURE 2:**
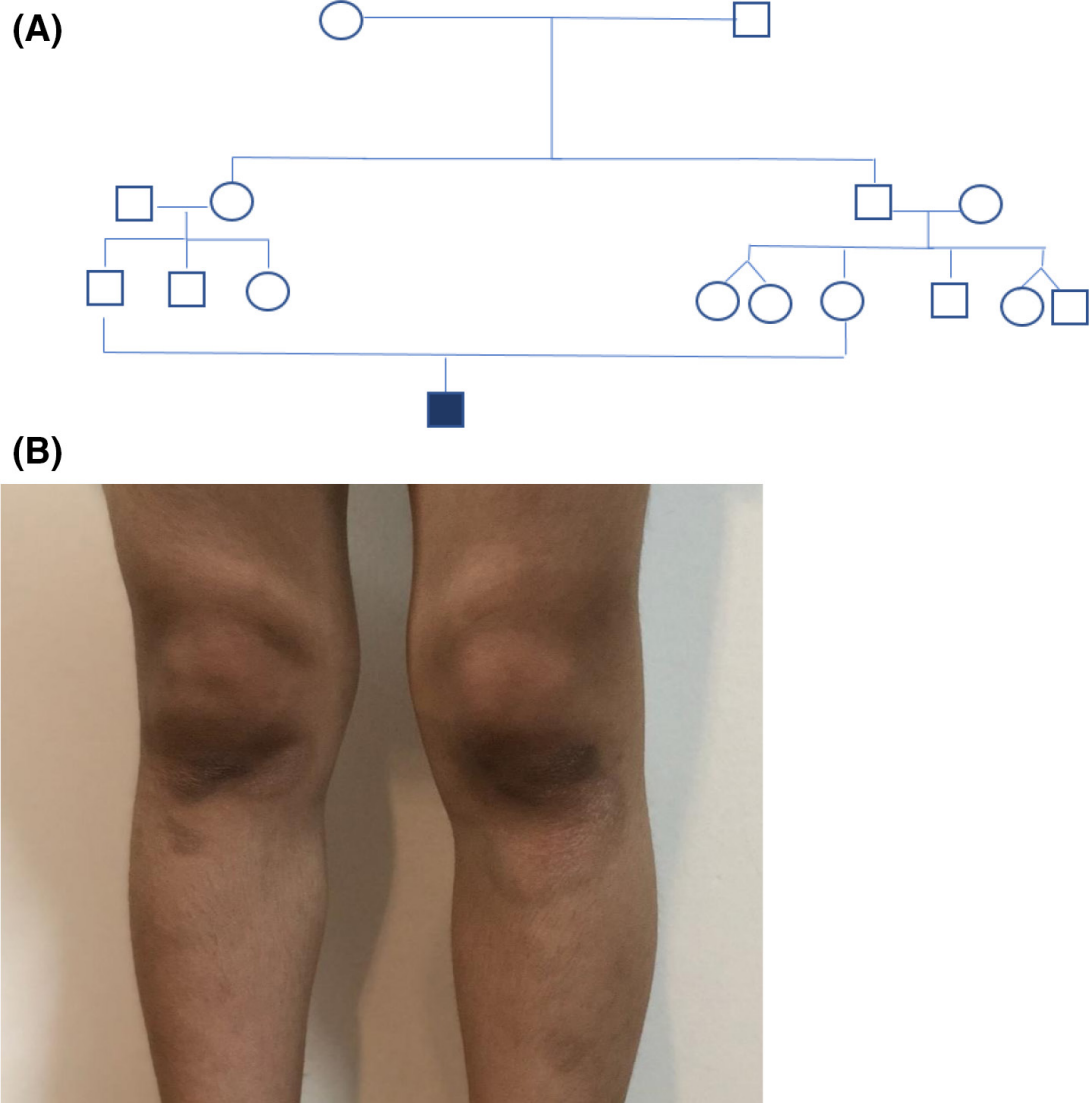
(A) Pedigree; (B) Brown spots appeared on the skin of a patient with ataxia‐telangiectasia (aged 4 years old). These spots also are visible on the elbows, ankles, and under the chin. Annotation: Healthy men □; healthy women ○; Boy with ataxia‐telangiectasia ■

At the age of 4 years, several brown spots appeared on the skin in the knees (Figure 2), elbows, ankle, and under the chin. Based on the pediatric immunologist diagnosis, the child was found allergic, especially to peanuts and potatoes.

As the child got older, he continued to put the sole of his left foot inward but did not fall down. Furthermore, the child exerted difficulty in painting and doing heels to toes (full tandem). Hence, his parents referred again to a pediatric neurologist at the age of 5.5 years.

Using a complete neurological examination with suspected cerebellar disorder, the child was referred for AT testing, including serum alpha‐fetoprotein (AFP) and AT‐related antibody concentrations (Table 1). Serum AFP concentration was elevated, whereas serum IgA level was decreased. These results were confirmed by genetic testing (ataxia‐telangiectasia mutated [ATM] class 1‐pathogenic; variant: Homozygous NM: 000051.3c.1369C>T (p. Arg457Ter)).

**TABLE 1:** Serum biochemical factors in a boy with ataxia‐telangiectasia aged 5.5 years old

Serum marker	Concentration
Blood group	A; RH positive
AFP (ng/ml)	127
Anti‐TTG (IgA), (U/ml)	0.5
IgG (mg/dl)	1097
IgA (mg/dl)	6
IgM (mg/dl)	99
IgE (CILA), (IU/ml)	1.1
Tetanus Ab (IgG), (IU/ml)	1.98
Pneumonia IgG (mg/L)	0.3
HBsAb (CILA), (mlu/ml)	116.20
CD results (40% LYMPH)	
CD 3	52.6
CD 4	28.4
CD 8	32.1
CD4/CD8	0.9
CD 16	23.3
CD 19	12.7

## 4 RESULTS

### 4.1 Supplements/physical therapy/oral dexamethasone

At the time of diagnosis (*i*.*e*., 5.5 years), the boy was vaccinated with the 13‐valent pneumococcal polysaccharide conjugate vaccine (*Prevenar*) in consultation with a pediatric immunologist.

**FIGURE 3:**
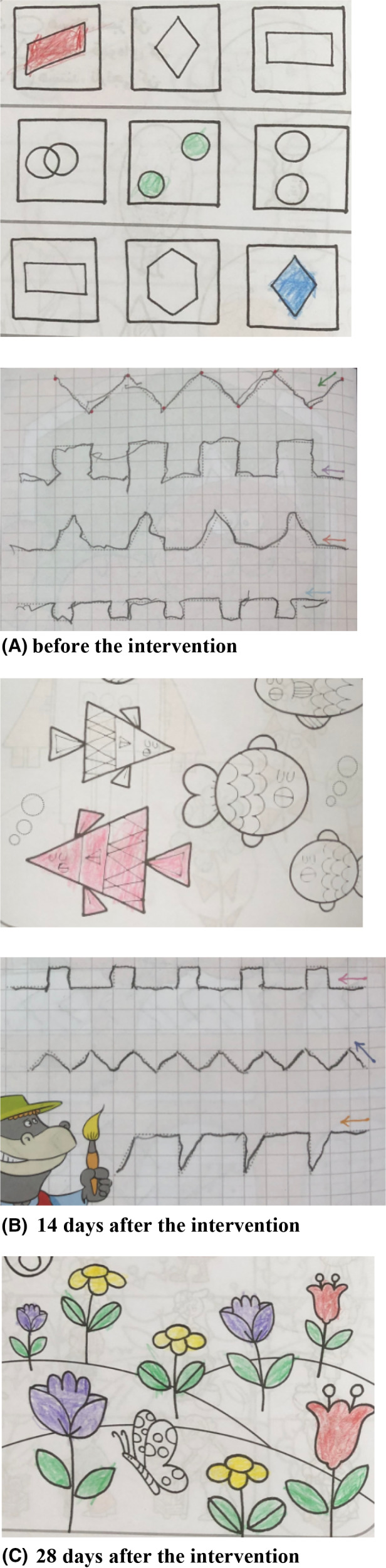
Effect of dexamethasone on child's painting and handwriting; (A) before the intervention; (B) 14 days after the intervention; (C) 28 days after the intervention

Physical therapy was initiated continuously for 3 times a week. Moreover, the patient received daily vitamin E (100 IU) supplement. Due to the lack of immune deficiency in this patient, oral dexamethasone was administered at a dose of 0.075 mg/kg/day {1.5 mg daily (0.5 mg for 3 times in 24 hours)} by the physician. The scale for the assessment and rating of ataxia (SARA) score was determined before (Score: 13) and 4 weeks after (Score: 6) the intervention (Table 2). The patient had an improvement of −7.0 SARA points after treatment over a period of 28 days. Dexamethasone also improved the child's painting and handwriting, as shown in Figure 3.

**TABLE 2:** Effect of dexamethasone on neurological symptoms by scale for the assessment and rating of ataxia (SARA)

Scale for the assessment and rating of ataxia (SARA)			
Variable/Score	Baseline	After 4 weeks intervention	After 8 weeks intervention
Gait	1	0	0
Stance	3	2	2
Sitting	1	0	0
Speech disturbance	1	1	1
Finger chase	1	1	1
Nose‐finger test	2	0	0
Fast alternating hand movements	2	1	1
Heel‐shin slide	2	1	1
Total score	13	6	6
Weight (kg)	18.8	20.2	21.0
Oral dexamethasone (mg per day)	1.5	1	0.5 to 0.0

### 4.2 Side effects

After 4 weeks, weight was increased by 1.4 kg, and thus dexamethasone dose was reduced to 0.05 mg/kg/day.

## 5 DISCUSSION

This is the first report assessing the effects of oral dexamethasone on neurological symptoms of AT in Iran. The SARA score was decreased by 7.0 points by oral dexamethasone therapy (0.075 mg/kg/day) over a period of 28 days.

In 2006, for the first time Buoni et al., reported the positive effects of betamethasone (0.1 mg/kg/day), a dexamethasone analog, on the neurological symptoms in a boy (3 years old) with AT after 14 days of treatment. However, side effects including increased appetite and weight, and moon face were observed after 28 days, leading to a change in therapy with methylprednisolone (2 mg/kg/day). This drug did not lead to beneficial effects that can be explained by the following reasons: 1. the anti‐inflammatory potency of methylprednisolone is 5 times lower than that of betamethasone, 2. the greater effect of betamethasone on the central nervous system, 3. different pathways of action of betamethasone and methylprednisolone, and 4. the particular effect of dexamethasone in increasing neuronal expression of 5‐lipoxygenase.^9^

In 2012, Zannolli et al. assessed the effects of oral betamethasone on ataxia symptoms among 13 children with AT (aged 5.8–16.1 years) in a multicenter randomized crossover trial using the International Cooperative Ataxia Rating Scale (ICARS). Taking betamethasone (0.1 mg/kg/day) over a period of 31 days lead to a reduction (by around 30%) in the ICARS total score.^7^

In 2014, Chessa et al. conducted a phase II randomized controlled trial with Ery‐Dex among 22 AT patients (aged 11.2  ±  3.5 years). Dexamethasone sodium phosphate content in the processed erythrocytes was 10.39 ± 6.28 mg/bag in females and 4.79 ± 4.68 mg/bag in male AT patients. Ery‐Dex significantly improved the neurological symptoms without any relevant steroid side effects.^10^

In 2017, Menotta et al., reported that when AT cell lines are treated with dexamethasone over a period of 48 hours, the AT cell membranes contents, cell components, and cell shape can be affected.^11^ In 2018, the underlying mechanisms of the dexamethasone's effects on 9 AT patients (age 6–12.8 years) were evaluated by Menotta et al., using CodeLink Whole Genome Bioarray. Ery‐Dex was administered for 6 months (at a dose of 13 ± 8.6 mg/bag). Dexamethasone modulated biologically compromised AT pathways and enhanced pathways involved in neuromodulation.^12^ In 2012, an *in vitro* study with lymphoblastoid cell lines (LCLs) found that a remarkable induction of a truncated protein, while maintaining kinase activity could partly explain the dexamethasone's impact on neurodegeneration in AT patients. Dexamethasone can potentially rescue the switch‐on of AKT, c‐RAF, and ERK1/2 in an ATM‐dependent manner and activate Chk1, which is a direct substrate of ATM. Dexamethasone can stimulate the AKT pathway. This drug is able to trigger a noncanonical splicing event in the ATM gene and generate a new type of ATMdexa1 transcription. ATMdexa1 can be converted to a functional protein that maintains the domain of native ATM kinase and gives the cell a second chance to produce a protein with reduced functions. This may also be one of the mechanisms by which dexamethasone exerts its beneficial effects.^13^, ^14^

The patient in the current case report had normal IgM levels (NIgM). A previous study reported that AT patients with NIgM have an extended survival comparing AT patients with elevated IgM levels (EIgM).^15^ Indeed, AT patients with EIgM are indicative of a more severe phenotype. Nevertheless, AT patients with NIgM have an augmented incidence of fatty liver or cirrhosis,^15^ and this should be considered by the physician.

## 6 CONCLUSION

Taking oral dexamethasone at a dose of 0.075 mg/kg/day for 28 days can improve ataxia symptoms in AT patients.

## AUTHOR CONTRIBUTIONS

MS and AS contributed to conceptualization. MS and MB contributed to investigation. MS contributed to writing—original draft. AS, TJ, and MB contributed to writing—review and editing.

## CONFLICT OF INTEREST

The authors declare that there is no conflict of interest.

## ETHICAL APPROVAL

The study protocol has been approved by the Ethics Committee of Mashhad University of Medical Sciences (IR.MUMS.REC.1400.128).

## CONSENT

Written informed consent was obtained from the patient to publish this report in accordance with the journal's patient consent policy.

## Data Availability

Data are available from the corresponding author upon reasonable request.
